# Impact of Optical Coherence Tomography (OCT) for Periodontitis Diagnostics: Current Overview and Advances

**DOI:** 10.3390/dj13070305

**Published:** 2025-07-04

**Authors:** Pietro Rigotti, Alessandro Polizzi, Anna Elisa Verzì, Francesco Lacarrubba, Giuseppe Micali, Gaetano Isola

**Affiliations:** 1 Unit of Periodontology, School of Dentistry, Department of General Surgery and Surgical-Medical Specialties, University of Catania, 95124 Catania, Italy; 2 Unit of Dermatology, Department of General Surgery and Surgical-Medical Specialties, University of Catania, 95124 Catania, Italy

**Keywords:** optical coherence tomography, periodontology, periodontitis, non-invasive imaging, periodontal diagnosis, artificial intelligence, periodontium

## Abstract

Optical coherence tomography (OCT) is a non-invasive imaging technique that provides high-resolution, real-time visualization of soft and hard periodontal tissues. It offers micrometer-level resolution (typically ~10–15 μm) and a scan depth ranging from approximately 0.5 to 2 mm, depending on tissue type and system configuration. The field of view generally spans a few millimeters, which is sufficient for imaging gingiva, sulcus, and superficial bone contours. Over the past two decades, its application in periodontology has gained increasing attention due to its ability to detect structural changes in gingival and alveolar tissues without the need for ionizing radiation. Various OCT modalities, including time-domain, Fourier-domain, and swept-source OCT, have been explored for periodontal assessment, offering valuable insights into tissue morphology, disease progression, and treatment outcomes. Recent innovations include the development of three-dimensional (3D) OCT imaging and OCT angiography (OCTA), enabling the volumetric visualization of periodontal structures and microvascular patterns in vivo. Compared to conventional imaging techniques, such as radiography and cone beam computed tomography (CBCT), OCT offers superior soft tissue contrast and the potential for dynamic in vivo monitoring of periodontal conditions. Recent advancements, including the integration of artificial intelligence (AI) and the development of portable OCT systems, have further expanded its diagnostic capabilities. However, challenges, such as limited penetration depth, high costs, and the need for standardized clinical protocols, must be addressed before widespread clinical implementation. This narrative review provides an updated overview of the principles, applications, and technological advancements of OCT in periodontology. The current limitations and future perspectives of this technology are also discussed, with a focus on its potential role in improving periodontal diagnostics and personalized treatment approaches.

## 1. Introduction

Periodontology is the branch of dentistry that focuses on the health of the supporting tissues of the teeth, namely the gingiva, alveolar bone, root cementum, and periodontal ligament [1]. The primary goal of periodontal treatment is to eliminate infection and resolve chronic inflammation to halt disease progression and prevent recurrence [2]. Periodontal diseases, such as gingivitis and periodontitis, are inflammatory conditions that affect these supporting tissues. In particular, periodontitis is characterized by the destruction of the gingiva, alveolar bone, cementum, and periodontal ligament [1], and the persistence of deep periodontal pockets (>5 mm) after active periodontal treatment is associated with an increased risk of disease progression and tooth loss [2]. On the contrary, peri-implant diseases, such as peri-implantitis and peri-implant mucositis, refer to pathological conditions affecting the tissues surrounding dental implants, similarly to how periodontal diseases affect natural teeth, and in these cases, the management and reconstruction of the interproximal papilla around implants is an important aspect [3].

The accuracy of diagnosing periodontal and peri-implant diseases is strongly reliant on radiographic examination, particularly for assessing alveolar bone levels, the morphology of bone defects, and overall bone quality. Radiographic imaging provides an objective and standardized method for evaluating hard tissues, minimizing the variability associated with manual clinical measurements and enhancing the precision of periodontal health assessment [4]. Radiographs are an essential component of periodontal evaluation for patients exhibiting clinical signs of periodontal destruction [5]. Conventional imaging techniques, such as intraoral radiographs, play a crucial role in periodontal diagnosis but have certain limitations when assessing complex periodontal bone defects [4]. Being two-dimensional, traditional radiographs may suffer from anatomical structure superimposition, distortions, and difficulties in accurately visualizing bone defect morphology [6,7]. To overcome these challenges, three-dimensional (3D) imaging techniques, such as cone beam computed tomography (CBCT), have been introduced to provide a more comprehensive understanding of periodontal structures [4,8]. CBCT generates high-resolution 3D images that can enhance diagnostic accuracy, particularly in evaluating infrabony defects and furcation involvement [6,9]. However, CBCT exposes patients to ionizing radiation, raising concerns regarding its routine use, especially for repeated follow-ups [10]. Additionally, CBCT images may be affected by artifacts caused by high-density materials within the scanning field, potentially compromising image quality [4].

An emerging non-ionizing modality is intraoral ultrasound (IU). This is a non-invasive and low-cost diagnostic tool widely used in medicine, which has demonstrated a great potential for intraoral imaging of the periodontium. Intraoral ultrasound imaging has shown good or excellent repeatability for measuring periodontal structures, such as alveolar bone crest to cementoenamel junction, gingival thickness (GT), and alveolar bone thickness [10]. It can measure GT without producing diagnostic images [11] and has shown a high correlation with direct measurements and with CBCT for facial bone levels around implants. Moreover, it also has the potential to evaluate vital structures relevant to dental implantology (lingual foramen artery, mandibular canal, maxillary sinus floor) and implant stability [12]. Although intraoral ultrasound shows promising results, further scientific validation and patient studies are needed to establish its accuracy compared to direct measurements [10,12].

Magnetic resonance imaging (MRI) is another non-ionizing technique that offers detailed 3D evaluations of soft tissues, including the gingiva, providing high tissue contrast. While valuable in diagnosing inflammatory diseases involving soft and hard tissues, its dental application is limited due to the accessibility of devices for dentists and artifacts caused by metallic dental materials affecting image quality. Nowadays, MRI’s reliability in diagnosing periodontal disease is known, aligning well with CBCT measurements and clinical assessments, showcasing its potential to improve diagnosis and treatment for periodontal diseases [4].

Finally, during the last years, within the imaging techniques field, many studies have been conducted about an innovative tool—optical coherence tomography (OCT). This is a non-invasive imaging modality that utilizes variations in light reflection to generate cross-sectional images of biological tissues, and essentially, it serves as an optical equivalent of ultrasound, employing near-infrared light instead of sound waves [11,13]. Compared to other imaging techniques, OCT provides superior resolution, allowing for the detailed visualization of tissue microstructures, and it has demonstrated significant potential in imaging key periodontal structures [14], detecting subgingival calculus and conducting precise morphometric analyses [15]. It has several advantages, such as non-invasiveness, high resolution, real-time imaging, and enhanced reproducibility. This means that OCT does not require ionizing radiation [11], delivers highly detailed images of both soft and hard tissues [15], and enables dynamic in vivo monitoring of periodontal conditions [10,16], and its non-contact nature improves the reliability of periodontal evaluations [10].

Despite the promising results reported in the literature, it is essential to recognize that OCT in periodontology is still a relatively new technique [17,18,19]. Actually, it has been reported that it offers micrometer-level resolution (typically ~10–15 μm) and a scan depth ranging from approximately 0.5 to 2 mm, depending on tissue type and system configuration. The field of view generally spans a few millimeters, which is sufficient for imaging gingiva, sulcus, and superficial bone contours [17].

Currently, its use is primarily confined to research settings, with limited clinical adoption due to factors such as instrument availability, portability, and ease of use, particularly for subgingival areas [19,20]. While numerous in vitro and ex vivo studies have demonstrated the potential of OCT in visualizing periodontal structures and measuring clinical parameters, such as sulcus depth and the presence of calculus [21,22,23,24,25,26], large-scale clinical evidence and longitudinal studies are still lacking, and these are necessary to fully validate its effectiveness in monitoring periodontal disease progression and evaluating treatment outcomes [19,20,27]. Therefore, many potential diagnostic and therapeutic applications of OCT in periodontology remain to be explored and validated through further research and clinical trials [21,28]; thus, the aim of this article is to provide a comprehensive review about the current research, uses, and main limitations of OCT.

## 2. Principles of Optical Coherence Tomography

**Figure 1:**
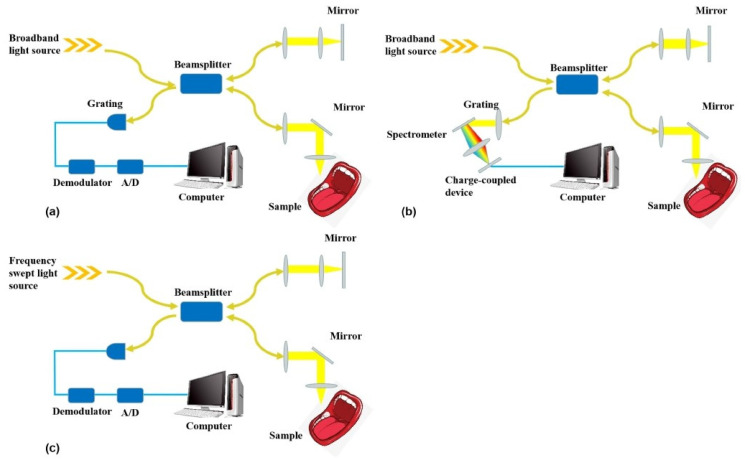
Schematic diagram showing the operating principle of TD-OCT (**a**), SD-OCT (**b**), and SS-OCT (**c**). From [17] with permission.

**Figure 2:**
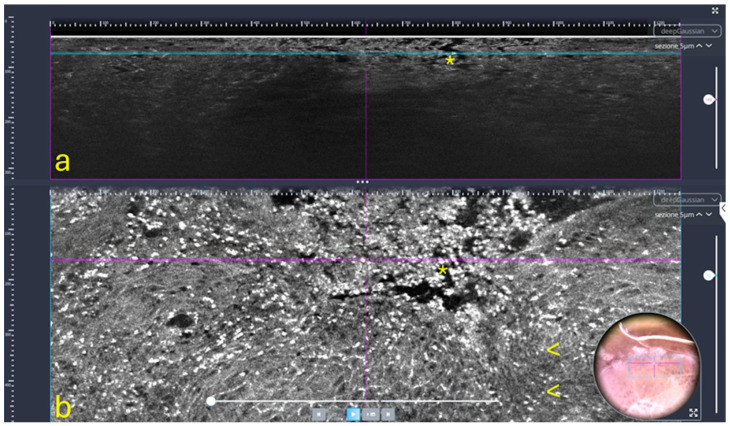
LC-OCT of the marginal interdental gingiva in a patient with periodontitis: vertical (**a**) and horizontal (**b**) sections showing epidermal disruption and erosions (*) and inflammatory cells (arrows). Insert: dermoscopic aspect of the examined area.

OCT is a non-invasive imaging technique that utilizes light to capture high-resolution images of biological tissue microstructures [13,29]. OCT operates on the principle of low-coherence interferometry, where a broad-spectrum light beam is directed onto the tissue [30]. Part of this light is reflected or backscattered by different tissue structures, while another portion is reflected by a reference mirror [31,32,33]. Interference occurs only when the optical paths of the backscattered and reference light match within the coherence length of the light source [31]. By measuring the intensity and time delay of the backscattered light echoes, it is possible to generate a detailed image of the tissue’s microarchitecture [29,34]. There are several types of OCT, including (Figure 1):

1. Time-domain OCT (TD-OCT): In this setup, the reference mirror is mechanically moved to scan tissue depth. Interference is recorded as a function of time, and an image is constructed point by point [30,35,36].
2. Fourier-domain OCT (FD-OCT): This technique captures the entire interference spectrum simultaneously using a spectrometer [24,37], and a Fourier transform is then applied to reconstruct the depth-resolved image [24,38]. FD-OCT provides significantly faster acquisition speeds compared to TD-OCT [24,37]. There are two main types of FD-OCT:
  ○ Spectral-domain OCT (SD-OCT): Instead of a photo detector, SD-OCT employs a spectrometer to capture the image. The spectrometer records the entire optical spectrum of the backscattered light, utilizing all wavelengths to extract detailed information about the esamine tissue, and a Fourier transform is subsequently applied to generate the image [30]. SD-OCT enables cross-sectional imaging in the Fourier domain by measuring both the intensity of backscattered or reflected light and its time delay. Compared to TD-OCT, SD-OCT achieves higher imaging speed due to its non-mechanical scanning mechanism, offers superior axial resolution, and has also found applications in ophthalmology, cardiology, and dermatology [29].
  ○ Swept-source OCT (SS-OCT): This technique employs a rapidly tunable laser that emits light at different wavelengths in quick succession, and the image is generated by analyzing the interference pattern as a function of wavelength variation [30]. Compared to SD-OCT, it has greater penetration depth, enhanced detection efficiency, extended imaging ranges, improved sensitivity with imaging depth, and dual-balanced detection capability [29].
3. Line-field confocal OCT (LC-OCT): This innovative technique is a recently developed non-invasive optical imaging technique designed for in vivo skin examination [39], which combines the principles of OCT and reflectance confocal microscopy (RCM), using line illumination and detection [40] (Figure 2). It provides high-resolution vertical images (B-scans), in real time from 8 to 10 frame/s, with an isotropic resolution of approximately 1 µm and a penetration depth of up to 500 µm [39,41,42]. LC-OCT is particularly well-suited for examining both healthy and pathological skin, enabling the visualization of cutaneous structures at the cellular level, including keratinocyte nuclei and the epidermal and dermal layers [39,41]. For this reason, it is used in the diagnosis, characterization, and therapeutic monitoring of various skin disorders, including benign and malignant skin tumors (such as melanoma, basal cell carcinoma, squamous cell carcinoma, and actinic keratosis), as well as inflammatory and infectious skin conditions [40]. AI and machine learning (ML) are emerging as essential tools for analyzing images obtained through LC-OCT and for detecting cutaneous anomalies. For instance, dedicated deep learning algorithms have been developed to assist in the analysis of these images, enabling the automatic segmentation of skin layers and keratinocyte nuclei. Moreover, AI, through convolutional neural networks, can assess the malignant potential of precancerous lesions, such as actinic keratoses (AK), by analyzing the undulation of the dermo-epidermal junction (DEJ) and quantifying cellular atypia [40].

Compared to other imaging techniques used in periodontology, such as CBCT, intraoral radiography, and ultrasound, OCT offers distinct advantages and limitations [43]. First of all, CBCT delivers high-resolution three-dimensional images of bone structures but involves exposure to ionizing radiation. In contrast, OCT does not use ionizing radiation and offers superior resolution for soft tissue imaging [43]. Secondly, intraoral radiography is a simple and cost-effective technique for assessing dental and bony structures; however, it provides limited resolution and two-dimensional images [43]. OCT, on the other hand, can generate high-resolution images of tissue morphology and has the potential for real-time monitoring of periodontal conditions [24]. Third, ultrasound uses sound waves to visualize soft tissues but has lower resolution than OCT and is not suitable for imaging hard tissues [29].

## 3. OCT Applications in Periodontology

In periodontology, accurate and early diagnosis is crucial for effective treatment and improved prognosis, yet current standard diagnostic methods present significant limitations. Traditionally, periodontal disease diagnosis relies heavily on periodontal probing and radiographic imaging. However, periodontal probing, a commonly used clinical test, is often questioned for its accuracy, with its reliability and reproducibility significantly reduced by factors such as probe thickness, applied pressure, tooth location, probe placement, and inherent periodontal irritation. Moreover, this procedure can cause considerable discomfort and pain to the patient, as the probe compresses soft tissue and may penetrate the gingival sulcus, damaging it [44]. It also struggles to accurately determine the precise coronal position of the connective tissue attachment [28].

To address these downsides regarding the periodontal diagnosis, OCT is emerging as a promising non-invasive, high-resolution imaging technique. Unlike traditional methods, OCT provides real-time, cross-sectional tomographic images of both hard and soft tissues with micrometric resolution, allowing for the detailed visualization of microstructures and enabling the early detection of subtle changes [19,26]. It offers direct, quantitative measurements of periodontal structures, such as pocket depth and gingival thickness, without contact or patient discomfort. This capability allows for frequent monitoring and the precise assessment of both hard and soft tissue changes, even in areas hidden from traditional visual or radiographic inspection [44]. Consequently, OCT holds significant potential for enhancing the diagnosis, monitoring, and assessment of periodontal disease treatments [19]. This section delves into the various applications of OCT in periodontology, focusing on the evaluation of periodontal tissue structures, detection of periodontal diseases, assessment of therapeutic outcomes, and monitoring of peri-implant tissues.

### 3.1. Assessment of Periodontal Tissue Structure

OCT has demonstrated effectiveness in visualizing and measuring key periodontal structures, including the gingiva, alveolar bone, and periodontal ligament [19].

#### 3.1.1. Gingiva

OCT enables the detailed imaging of gingival structures and oral mucosa in vivo [23,45] (Table 1). Studies have shown its capability to distinguish the oral epithelium, sulcular epithelium, connective tissue, and dentogingival junction and to measure gingival thickness and sulcus depth in a non-invasive way [46,47]. In a pilot study, Fernandes et al. assessed periodontal structures and sulcus depth in healthy volunteers using a 1325 nm SS-OCT system, comparing the results with conventional periodontal probing. OCT imaging successfully identified relevant anatomical regions of the dentogingival complex [23]. Mota et al. employed two Fourier-domain OCT systems operating at different wavelengths (930 nm and 1325 nm) for the structural analysis of porcine periodontal tissue, effectively visualizing distinct periodontal structures and measuring gingival thickness and sulcus depth [47]. Kakizaki et al. investigated the effectiveness of OCT for the in vivo assessment of periodontal tissue profiles in healthy volunteers, measuring gingival and epithelial thickness, alveolar bone crest position, and biologic width [46]. Di Stasio et al. further defined normal epithelial thickness values at different oral cavity sites using OCT [45]. Additionally, OCT imaging can reveal microstructural details, such as epithelial ridges and gingival vasculature, through OCT angiography (OCTA) [48].

#### 3.1.2. Alveolar Bone

OCT provides surface-level insights into alveolar bone morphology and its variations [49]. Although its penetration depth in hard tissues is limited compared to radiographs or CBCT, OCT can visualize the alveolar bone crest near the gingival surface [24,27,46]. In an in vitro study, Chang W.-T. et al. demonstrated that a 1310 nm SS-OCT system could accurately and non-invasively measure the crestal bone level (CBL) through artificial gingiva, enabling the semi-automated identification of critical periodontal landmarks. Their findings showed minimal deviation from reference values, indicating OCT’s potential for monitoring subtle bone changes [27]. Kakizaki et al. also successfully identified alveolar bone crest positions in OCT images of healthy volunteers [46]. Furthermore, an OCT system incorporating deep neural networks has been developed for the automated and quantitative measurement of alveolar bone levels [49].

#### 3.1.3. Periodontal Ligament

The direct visualization of the periodontal ligament using OCT can be challenging due to its anatomical location and limited thickness. However, preliminary studies have explored OCT’s potential in assessing the periodontal ligament under specific conditions, such as during orthodontic movement. Baek et al. conducted a preliminary study using OCT to observe the periodontal ligament during orthodontic tooth movement, suggesting OCT’s applicability in this field [25,50].

### 3.2. Detection of Periodontal Diseases

OCT offers various potential applications for detecting periodontal diseases, including the visualization of dental calculus and evaluation of gingival inflammatory status [51].

Periodontitis is closely associated with the presence of dental calculus [52], and OCT has demonstrated its ability to detect supragingival and subgingival calculus in in vitro and ex vivo studies [24,53]. In an in vitro study, Tsubokawa et al. evaluated the efficacy of SS-OCT in detecting calculus and root cementum during periodontal therapy, clearly visualizing calculus as an amorphous white-gray structure on the root surface [53]. Hsieh et al. reported that SS-OCT imaging provided the near real-time visualization of dental calculus in vitro [54]. Park et al. compared the diagnostic accuracy of OCT, micro-CT, and histology in periodontal disease, confirming OCT’s capability to detect both supragingival and subgingival calculus [24]. Mota et al. observed that the presence of calculus increased the distance between the tooth and sulcular epithelium, making a widened space clearly identifiable in OCT images [47]. Beyond calculus detection, OCT can contribute to periodontal disease diagnosis by measuring pocket depth and visualizing attachment loss [49]. Kim et al. reported improved accuracy in periodontal pocket depth assessment using OCT on a porcine model, demonstrating that OCT could effectively visualize periodontal pockets and detect attachment loss, with pocket depth measurements comparable to manual probing [26]. Moreover, Surlin et al. found that OCT can evaluate gingival inflammatory status by analyzing pixel density variations in OCT images [55], and they used OCT to examine inflammatory changes in the gingival tissue of periodontal patients with non-alcoholic fatty liver disease (NAFLD), suggesting NAFLD as a potential aggravating factor for periodontal inflammation [56].

### 3.3. Evaluation of Periodontal Therapy Outcomes

OCT has shown promise as a tool for assessing the outcomes of various periodontal therapies by providing insights into tissue healing and reductions in calculus and biofilm accumulation [22].

#### 3.3.1. Non-Surgical Periodontal Treatment

OCT can be utilized to monitor the effectiveness of non-surgical periodontal treatment by evaluating subgingival calculus removal, as reported in a clinical study carried out by Tsubokawa et al., who obtained OCT images before and after the non-surgical periodontal treatment in human patients, observing the disappearance of calculus in post-treatment scans [53]. Fernandes et al. tracked periodontal disease patients with OCT, noting reductions in gingival thickness associated with calculus removal, along with improvements in probing depth and treated region imaging [22].

#### 3.3.2. Regenerative Procedures

OCT has been employed to monitor gingival tissue regeneration following periodontal plastic surgery. About this, Fernandes et al. used OCT to track tissue repair in patients undergoing esthetic periodontal plastic surgery over a 60-day period, observing different healing phases at 15 and 60 days postoperatively [57]. In a case report, Graça et al. described OCT as a non-invasive diagnostic and follow-up tool for evaluating gingival recovery after esthetic oral rehabilitation, demonstrating its ability to visualize new sulcus formation and close gingival adaptation to the tooth post-treatment [58].

Furthermore, OCT’s capabilities extend to additive procedures and detailed wound healing monitoring. It is valuable for measuring gingival thickness and soft tissue dimensions, aiding in planning and evaluating outcomes of gingival augmentation, root coverage procedures, and connective tissue grafts, including identifying palatal vessels for bleeding control at donor sites [18,24,44]. For wound healing, OCT provides real-time, high-resolution cross-sectional images, allowing for the assessment of wound epithelization, inflammation, lesion progression, and overall healing extent over time. It can depict inflammatory tissues and their resolution, changes in gingival thickness and volume, and alterations in pixel density indicative of inflammation [18]. Moreover, advanced techniques like OCTA enable visualization and quantification of blood vessels, facilitating assessment of angiogenesis and inflammation resolution, which is particularly beneficial for delicate surgeries and long-term follow-up [59]. Despite these advantages, a key limitation of OCT is its limited penetration depth, typically 1.5 mm in human gingiva, which can impact the imaging of deeper structures [51].

Looking ahead, OCT holds significant promise for the long-term assessment of remaining periodontal tissues, particularly in maintenance patients. Standard periodontal treatment, such as scaling and root planing, is foundational and aims to achieve a smooth, clean, and biologically acceptable root surface, free from bacterial plaque and calculus. This clean surface is essential for the adhesion of fibroblasts, which is critical for periodontal regeneration. Given this crucial requirement, OCT’s non-invasive and high-resolution imaging capabilities could prove invaluable for ongoing monitoring. It could facilitate the assessment of root surface conditions to detect any residual calculus or biofilm and to evaluate the overall health and integrity of regenerated or maintained periodontal tissues, thereby contributing to the long-term success of periodontal therapy as evidenced by improvements in clinical attachment levels and reductions in probing pocket depths [60].

### 3.4. Monitoring of Peri-Implant Tissues

Monitoring peri-implant tissues is crucial for preventing and managing peri-implant diseases, such as peri implant mucositis and peri-implantitis [51], and OCT holds potential for the non-invasive assessment of these tissues [61] (Table 2). A preliminary study by Sanda et al. investigated OCT’s effectiveness in implantology, showing that implants covered by mucosa thinner than 1 mm could be clearly identified in OCT images. Specifically, the surface of the implant body underneath the mucosa was revealed in samples with mucosal thicknesses ranging from 0.35 mm to 1.11 mm. Submucosal objects could generally be detected if the mucosa thickness was less than 2.5 mm. However, it was challenging to capture clear images of the implant body and alveolar bone when implants were embedded in pig jawbones, as only the surface of the mucosa was typically shown. Excess cement residues around implants were visible in samples with mucosal thicknesses below 3 mm. More precisely, cement remnants in the submucosal area could be detected by OCT if the sulcus depth was less than 2 mm and the thickness of the mucosa was less than 3 mm [61]. Kim et al. used OCT to visualize and quantitatively measure peri-implant bone defects in porcine mandibles, finding a strong correlation between OCT and digital caliper measurements. In OCT images, the portion of the implant fixture surrounded by bone was not visible, but the threads of the fixture within a bone defect were. This allowed for the measurement of peri-implant bone loss by calculating the length of these visible threads, knowing the physical pitch of the implant. The average defect depth measured by OCT was 5.11 ± 1.33 mm, which was very close to the 4.88 ± 1.28 mm measured by a digital caliper. Although OCT measurements were, on average, 0.23 mm greater, this was considered clinically insignificant. The slight difference might be due to the optical beam penetrating very thin bone areas that were sufficiently transparent, which OCT then interpreted as bone loss. The reliability of OCT measurements showed high agreement with caliper measurements, indicated by a high intraclass correlation coefficient and a Bland–Altman analysis, where most measured values fell within the 95% confidence interval [62]. OCT may also facilitate the early detection of peri-implantitis [51]; however, researchers emphasize that current OCT imaging applications for implants remain limited [61].

## 4. Advancements and Technological Innovations

The evolution of OCT has introduced transformative capabilities in the field of periodontology, overcoming the limitations of traditional diagnostic techniques [23,47]. It is very important to highlight the key advancements and technological innovations shaping the application of OCT in diagnosing and managing periodontal diseases, focusing on high-resolution and 3D imaging capabilities, the integration of AI and ML, portable and handheld OCT devices, and the potential for real-time chairside diagnostics.

### 4.1. High-Resolution 3D Imaging Capabilities

OCT stands out as a non-invasive biomedical imaging technique that utilizes near-infrared coherent light to provide high-resolution cross-sectional visualizations of biological samples [63,64]. Its ability to generate high-resolution (on the micron scale) and high-contrast images of intraoral structures offers a significant advantage over standard imaging modalities [65,66]. In periodontology, this translates into the ability to visualize microstructural details of gingival and periodontal tissues, including the gingival epithelium, sulcus depth, and variations in connective tissue structure [63,65].

Additionally, OCT can provide real-time 3D tomographic images of tissues. This three-dimensional imaging capability allows for a more comprehensive assessment of periodontal pockets, gingival morphology, and soft-to-hard tissue interfaces. For instance, Mota et al. analyzed the structure of periodontal tissues in a porcine model using two Fourier-domain OCT systems with wavelengths of 930 and 1325 nm, demonstrating the ability to identify free and attached gingiva and to non-invasively measure gingival thickness and sulcus depth. Their findings suggested that OCT systems operating at 1325 nm offer superior performance due to greater tissue penetration [47,63].

The high-resolution and 3D visualization capabilities of OCT are fundamental for the early diagnosis of periodontal diseases, monitoring disease progression, and evaluating treatment efficacy [65]; indeed, OCT imaging can reveal microstructural changes that may not be evident during traditional clinical examinations or with two-dimensional radiographs [65,66].

### 4.2. Integration of Artificial Intelligence and Machine Learning

**Figure 3:**
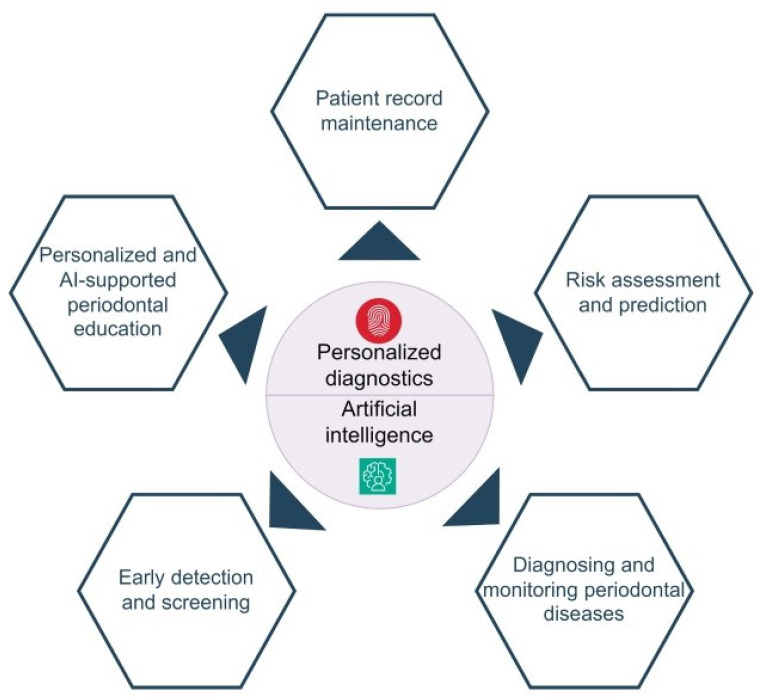
Scope of AI-based personalized diagnostics in the field of periodontology. From [67] with permission.

The integration of AI and ML with OCT is opening new frontiers in the diagnosis and management of periodontal diseases [65,67] (Figure 3). AI can be applied to large OCT datasets to automate the detection of pathological features, enhance diagnostic accuracy, and provide prognostic insights [67,68].

It was explored the use of ML algorithms, particularly convolutional neural networks (CNNs), for analyzing periodontal OCT images. These algorithms can be trained to automatically segment gingival tissues, identify the cementoenamel junction (CEJ), and detect periodontal bone loss (PBL) lesions based on radiographic images [69]. Although AI applications in periodontology remain relatively limited, some studies have investigated its potential in various diagnostic tasks, such as evaluating panoramic and periapical radiographs to detect periodontal bone loss, stage and classify periodontitis, and identify dental implant types [67].

In the specific context of OCT and oral diseases, including periodontitis, AI has demonstrated great potential in enhancing diagnostic accuracy. AI can assist in OCT image interpretation by identifying subtle morphological changes that might be overlooked by the human eye. For instance, AI-based algorithms can be used to quantify epithelial thickness, assess the regularity of the epithelial–connective junction, and detect inflammatory features in gingival tissues [65].

James et al. conducted an in vivo study using an artificial neural network and an algorithm-based scoring system to identify malignant/dysplastic lesions in 232 patients, achieving a sensitivity of 95% for dysplastic lesion differentiation and 93% for malignant lesions [70]. They later confirmed the ability of a retrained convolutional neural network to distinguish 3D OCT images of abnormal mucosa in the head and neck region, with a sensitivity of 100% and a specificity of 70% [17].

The integration of OCT with AI and ML holds great promise for significantly improving the efficiency and accuracy of periodontal diagnostics, providing clinicians with a powerful tool for early detection and personalized disease management [67,71]; however, it is crucial to consider the need for human supervision in clinical decision making [72].

### 4.3. Portable and Handheld OCT Devices

Advancements in OCT system miniaturization have led to the development of portable and handheld devices, further expanding the potential applications of this technology in periodontology [65,73,74]. These compact devices offer several advantages, including ease of use, accessibility to various areas of the oral cavity, including posterior teeth and buccal and lingual surfaces, and potential use in point-of-care settings [66,74].

Traditional commercial OCT systems are often bulky and not specifically designed for oral imaging, limiting accessibility primarily to frontal views of anterior teeth, but the development of customized handheld probes and portable OCT systems has overcome some of these limitations [66]. These devices often integrate compact light sources, miniaturized scanning systems, and portable data processing units [74].

Won et al. introduced a portable spectral-domain OCT system based on a handheld probe, which was developed and demonstrated for imaging dental plaque and gingiva in a clinical setting. Trained dental hygienists used the handheld OCT probe on human subjects with mild to moderate gingivitis and sufficient dental plaque, exploring the feasibility of OCT imaging for both anterior and posterior teeth, as well as buccal and lingual surfaces. Additionally, the longitudinal tracking of dental plaque was performed to observe the effects of brushing products [66].

The introduction of portable and handheld OCT devices marks a significant step toward the broader adoption of this technology in daily dental practice [65], and thanks to their portability and ease of use, they can be particularly suitable for chairside imaging and potential applications in screening and monitoring [73].

### 4.4. Potential for Real-Time Chair-Side Diagnostics

The ability of OCT to provide high-resolution real-time imaging, combined with the availability of portable and handheld devices, opens exciting prospects for chairside diagnostics in periodontology [65,66]. Real-time imaging enables clinicians to immediately visualize the microstructures of periodontal tissues during a dental visit, potentially improving diagnostic and therapeutic decision making [65].

Chairside OCT could overcome some limitations of traditional diagnostic techniques. For instance, periodontal probing depth measurement, while considered the gold standard, remains an analog technique in a digital era, subject to inter- and intra-operator variability and influenced by site-specific factors. OCT, instead, offers the possibility of a non-invasive and objective assessment of periodontal pockets and surrounding soft tissues [63].

**Figure 4:**
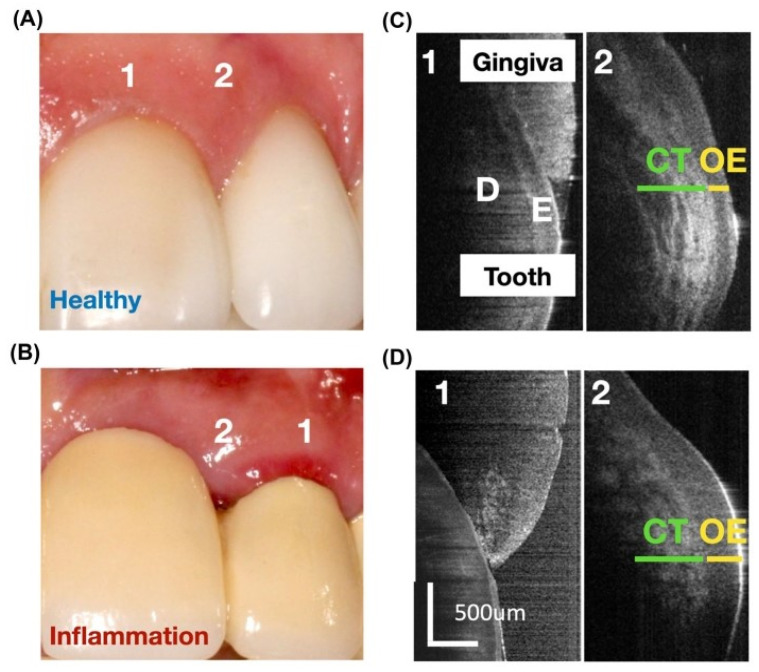
In vivo imaging of marginal gingiva in anterior maxilla. Representative sites of healthy gingiva and gingivitis around a natural tooth with healthy gingiva (**A**) and a prosthetic crown with marginal gingivitis (**B**) at one mid-facial (1) and one interproximal (2) sites. (**C**,**D**) μOCT imagining of the healthy (top) and gingivitis lesion (bottom) showing soft tissue compartments, the connective tissue, and oral epithelium. D, dentin; E, enamel. From [28] with permission.

**Figure 5:**
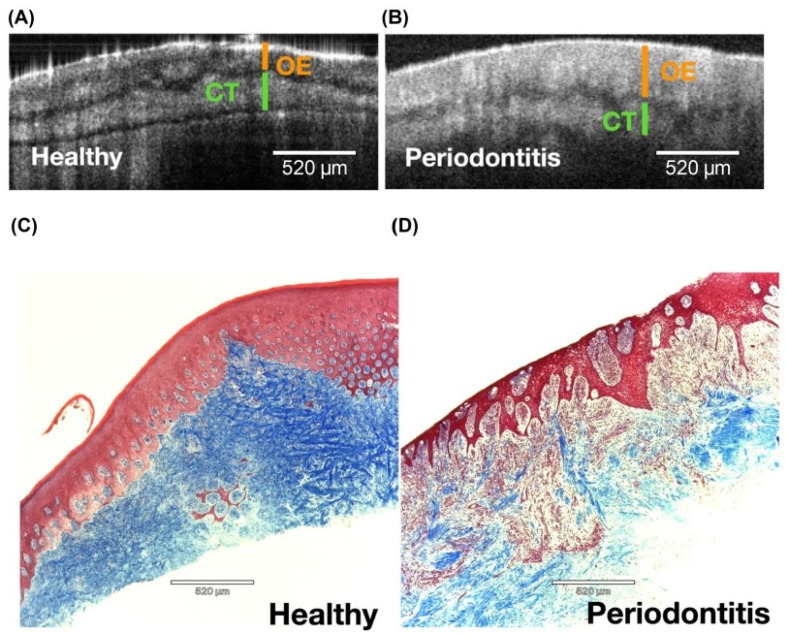
μOCT-histology correlative analysis of epithelium and connective tissue compartments of human gingiva. Representative μOCT images of excised gingival samples depicting oral epithelium (OE) and connective tissue (CT) compartments in health (**A**) and periodontitis (**B**). The corresponding Masson trichrome stained slides showing the OE (pink/red) and CT (blue, collagen) in health (**C**) and periodontitis (**D**). Significantly less collagen and more cell infiltration are noted in CT of periodontitis lesion scale bar, 520 μm. From [28] with permission.

Additionally, the combination of OCT with other chairside imaging technologies, such as Raman microspectroscopy (RMS), could provide complementary information on the biochemical composition and morphology of periodontal tissues. A pilot study developed and tested a combined RMS/micro-OCT (μOCT) approach for chairside quantification of gingival collagen, DNA, epithelium, and connective tissue, demonstrating its ability to differentiate between healthy and inflamed periodontal sites [28] (Figure 4 and Figure 5).

The potential for real-time chair-side diagnostics with OCT could lead to the earlier detection of periodontal diseases, more personalized treatment plans, and more effective monitoring of therapeutic responses. Non-invasive, real-time imaging could also enhance patient comfort and reduce the need for invasive diagnostic procedures [65].

## 5. Challenges and Limitations

Despite the significant advancements and promising advantages of OCT in biomedical imaging, as well as its growing potential in periodontology, several challenges and limitations hinder its broader adoption and full integration into daily clinical practice [15,17].

One of the main barriers is cost and accessibility. OCT systems, particularly those equipped with advanced features and high-resolution capabilities, require a considerable initial investment, limiting their accessibility for private dental practices or institutions with restricted financial resources [75]. Although efforts are being made to develop low-cost and portable OCT systems with promising applications in ophthalmology and other medical fields, the availability of such solutions specifically adapted to periodontology remains limited [75]. The need for specialized optical probes and the associated maintenance costs further impact the feasibility of widespread adoption by dental professionals [15,17].

Another crucial aspect concerns operator training and the standardization of procedures. Acquiring and interpreting OCT images require specific expertise and a deep understanding of the instrument’s operating principles, as well as the morphological characteristics of periodontal tissues [15]. Similar to what has been observed in ophthalmology for OCT scan interpretation, dental professionals need proper training to capture high-quality images and correctly interpret results, distinguishing artifacts from actual pathological features [76,77]. Moreover, the lack of standardized protocols for image acquisition, data analysis, and reporting can lead to significant inter-operator and intra-operator variability, limiting the comparability of data obtained across different studies and clinical settings [17,78,79]. Thus, standardizing imaging methods, operational parameters, and analysis criteria is essential to ensure the reproducibility of measurements and the clinical validity of OCT in periodontology [78].

An inherent limitation of OCT is its restricted penetration depth compared to other imaging techniques. OCT relies on detecting light backscattered from the tissue, and the strong scattering and absorption of light in biological tissues, especially in denser or inflamed tissues like bone, limit the imaging depth to approximately 0.4 to 2.0 mm [17]. While this depth is sufficient for visualizing the superficial structures of periodontal soft tissues, the tooth surface, and potentially the superficial alveolar bone crest, it may not be adequate for assessing deeper periodontal lesions, infrabony defects, or detailed bone architecture [50]. OCT faces limitations particularly in dense bone. This is primarily due to the high multiple scattering that occurs in such highly scattering media, like bone and cartilage, which severely restricts the imaging depth [80]. In such cases, techniques like conventional radiography, CBCT, or ultrasound may offer significantly greater penetration [15,81]; thus, this depth limitation may restrict the clinical applications of OCT in scenarios that require a detailed evaluation of deeper periodontal structures.

Finally, regulatory implications and clinical validation for the use of OCT in periodontology must be considered. The introduction of a new imaging technology into clinical practice requires approval from regulatory bodies and robust clinical evidence demonstrating its efficacy, safety, and added value compared to existing diagnostic and monitoring techniques [17,82]. Although OCT is already approved and widely used in several medical applications, such as ophthalmology and dermatology [17], its specific application in periodontology may require additional well-designed clinical studies conducted on a large patient cohort to validate its diagnostic and prognostic utility, as well as its role in guiding therapeutic decisions for periodontal diseases.

## 6. Future Perspectives

One of the most promising developments concerns its potential applications in personalized periodontal treatment. Traditional approaches to periodontal care often follow a “one-size-fits-all” strategy, which may not account for the complex interplay of genetic, environmental, and behavioral factors that influence disease progression in each individual [67]. OCT, with its capacity for non-invasive and in vivo assessments of the microstructural characteristics of periodontal tissues, could provide valuable insights for stratifying patients based on their risk of disease progression and their potential response to different treatments [83]. For instance, using the axial resolution of the images, OCT could be used to detect subtle early changes in epithelial morphology and gingival microvascularization that may predict future clinical attachment loss [17,84]. The ability to precisely quantify tissue parameters, such as epithelial thickness, connective tissue density, and gingival sulcus depth, through OCT could enable the real-time monitoring of individual patient responses to personalized therapies, such as photobiomodulation or localized drug applications [83,85]. Moreover, integrating OCT with salivary biomarker analysis, reflecting the patient’s inflammatory status and genetic susceptibility, could create a comprehensive diagnostic framework to guide targeted and predictive therapeutic decisions, and the development of at-home test kits based on salivary biomarker analysis, supported by AI algorithms, could further empower patients in managing their periodontal health [67,86]. Moreover, future studies could assess OCT reliability in other conditions predisposing to periodontal inflammation, such as orthodontic treatment, which may favor specific inflammatory patterns [87].

**Figure 6:**
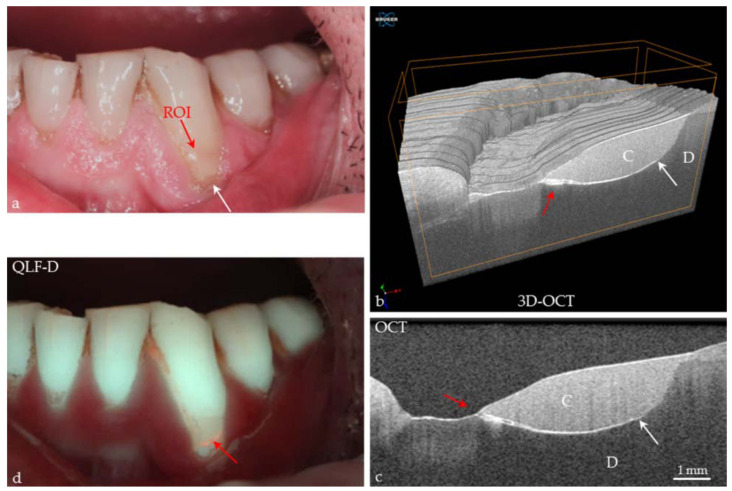
Canine tooth in vivo. (**a**) A cervical composite restoration (C) shows a margin of discoloration (white arrow); (**b**,**c**) in the 3D and 2D OCT images, a margin gap with an ingress of material is detected (red arrows). At dentin (D), an extensive interfacial gap has formed (white arrows); (**d**) using quantitative light-induced fluorescence, the image shows red fluorescence (red arrow) at the cervical restoration margin, indicating bacterial activity by porphyrin invasion into the gap. There are no signs of “secondary caries” at the restoration margin, but the stability of the situation requires observation over time (monitoring). From [88] under the terms of Creative Commons Attribution (CC BY) license.

In addition, recently, 3D OCT has been employed in dentistry [88] (Figure 6) and could be further analyzed for periodontal tissues.

The future of periodontal diagnostics will likely embrace multimodal imaging approaches, where OCT is integrated with other techniques to overcome its inherent limitations, such as penetration depth [30]. Combining OCT with ultrasonography could provide complementary information on soft tissue structures and the presence of deeper abscesses or lesions [89]. Integration with digital radiographic techniques, such as intraoral radiography and CBCT, supported by AI-driven image segmentation and registration algorithms, could offer a comprehensive view of both tissue surfaces and underlying bone structures; for instance, OCT could be used to assess soft tissue healing following regenerative surgery, while CBCT visualizes bone regeneration [4]. The combined use of OCT with functional imaging techniques, such as polarization-sensitive OCT, could provide information on birefringence and collagen fiber orientation, helping to characterize gingival tissue quality and its response to treatment [90]. Furthermore, the development of OCT probes with angiographic capabilities would allow for the visualization of gingival and peri-implant microcirculation, offering early indicators of inflammation and therapeutic response [15,17].

AI-assisted analysis could play an important role in expanding the clinical applications of OCT. For instance, the analysis of OCT images of oral tissue can be challenging and time-consuming; however, collaboration with AI has significantly reduced the workload and decreased subjective factors [17]. AI can also automate the analysis of the large volumetric datasets acquired with OCT, enabling faster and more objective quantification of tissue parameters [17]. Deep learning algorithms, such as convolutional neural networks (CNNs), have demonstrated high accuracy in distinguishing between healthy and pathological tissues, identifying malignant and dysplastic lesions [17], and automatically segmenting different periodontal structures in OCT images [4]. Moreover, AI can help standardize OCT image interpretation, reducing inter-operator variability and improving diagnostic accuracy [4], and the development of AI-based decision support systems that integrate OCT data, clinical information, and patient history could assist clinicians in making more precise diagnoses, planning personalized treatments, and predicting prognoses [4,83]. The application of natural language processing (NLP) to the analysis of electronic health records and patient feedback could further enrich the clinical picture, enhancing communication and patient satisfaction [67]. Additionally, AI could be utilized to develop self-monitoring tools based on intraoral images captured by patients, providing personalized feedback and alerting professionals when necessary [67,86].

For the effective clinical implementation of OCT in periodontology, several challenges need to be addressed [30]. First, the development of more compact, ergonomic, and oral-specific OCT probes is crucial to enable rapid and stable image acquisition in all areas of the oral cavity, overcoming the limitations of current probes, which are often bulky and difficult to stabilize [17]. Reducing the cost of OCT systems and developing portable, low-cost devices will make this technology more accessible to a larger number of dental practices [82]. A key aspect is the integration of OCT into undergraduate and postgraduate dental curricula to ensure adequate training in its use and interpretation [30], and also, the development of standardized clinical protocols for OCT image acquisition and analysis in different periodontal conditions is essential to ensure the reproducibility and clinical validity of results [4]. Conducting large-scale and well-designed clinical studies is necessary to validate the diagnostic and therapeutic efficacy of OCT and AI in periodontology and to demonstrate its added value compared to traditional techniques [91,92,93]; thus, collaboration among researchers, clinicians, manufacturers, and regulatory agencies will be fundamental in overcoming regulatory barriers and ensuring the safe and effective adoption of OCT in daily clinical practice, ultimately aiming to improve periodontal health and patient quality of life [67,82,92]. In conclusion, the future prospects for the application of OCT and AI in periodontology are extremely promising; their integration offers the potential to revolutionize early diagnosis, personalized treatments, therapeutic monitoring, and the long-term management of periodontal diseases, paving the way for a new era of precision dentistry [67,83,94]. By overcoming current challenges through research, technological innovation, and interdisciplinary collaboration, the full potential of these technologies can be harnessed to improve both oral and systemic health in patients [86].

## 7. Conclusions

OCT represents a significant step forward in periodontal diagnostics, offering non-invasive, real-time, high-resolution imaging of both soft and hard oral tissues. This review has shown that OCT can delineate gingival microstructures, identify subclinical inflammatory changes, and track soft-tissue regeneration with detail well beyond that of conventional probes and radiographs. It also excels at visualizing subgingival calculus and early crestal bone alterations, supporting the more precise assessment of disease activity and treatment response. Despite challenges, limited penetration depth, high equipment costs, and operator learning curves, OCT’s dynamic, quantitative outputs (tissue thickness, pocket geometry, microvascular flow) position it uniquely for personalized periodontal care. Integrating AI further promises to automate segmentation, reduce observer variability, and deliver decision-support analytics that stratify patient risk and tailor therapy.

### Future Directions

Looking ahead, the most promising research and development avenues include the creation of compact, cost-effective, chairside OCT probes optimized for difficult intraoral angles; the fusion of OCT with complementary modalities, such as CBCT, OCTA, or spectroscopic imaging to yield multiparametric maps of anatomy and inflammation; and the training of deep-learning models on large, annotated clinical datasets to enable instantaneous, automated interpretation. Standardized scanning protocols and large-scale, prospective clinical trials will be essential to validate OCT’s diagnostic accuracy, demonstrate its impact on treatment outcomes, and support its adoption into everyday periodontal practice. By advancing along these paths, hardware miniaturization, multimodal integration, AI-driven analysis, and rigorous clinical validation, OCT can evolve from a powerful research tool into a routine, transformative component of precision periodontal care.

## Figures and Tables

**Table 1:** Studies assessing the potential use of optical coherence tomography to evaluate health and pathology of periodontal tissues. From [11] with permission.

Study	Parameters Measured	Sample	Tool	Main Findings Between Modalities
Kakizaki et al. (2018) [46]	Thickness of gingiva, mucosa, and biologic width	177 lower anterior teeth of 30 periodontally healthy patients	Dental swept-source OCT system (Prototype 2, Panasonic, Ehime, Japan); 1330 nm central wavelength laser source with 100 nm bandwidth at a scanning rate of 30 kHz	Demonstrated that OCT could visualize and measure the thickness of gingiva, mucosa, and biologic width
Fernandes et al. (2017) [23]	Gingival sulcus depth	All anterior teeth of 23 periodontally healthy patients, with a total of 445 buccal examination sites	Swept-source OCT system (model unspecified, Thorlabs, Newton, United States); 1325 nm central wavelength laser source with 100 nm bandwidth at a scanning rate of 16 kHz	1. Mean buccal gingival sulcus depth measured by OCT < manual probing and automated probing with Florida Probe by 0.57 mm and 0.39 mm, respectively (*p* < 0.001) 2. Time needed to obtain OCT images >manual probing and automated probing by 17.84 min and 17.17 min, respectively (*p* < 0.001)

**Table 2:** Nonclinical studies assessing the potential use of optical coherence tomography to evaluate the health and pathology of peri-implant tissues. From [11] with permission.

Study	Parameters Measured	Sample	Tool	Main Findings Between Modalities
Kim et al. (2018) [62]	Peri-implant bone defect	15 implants were placed in 4 dead porcine mandibles; 75 bone defects were prepared	Swept-source OCT system (model unspecified, Oztec, Daegu, Korea); 1310 nm central wavelength laser source at a scanning rate of 50 kHz	Bone defect depth measured by OCT > caliper by 0.23 mm (*p* < 0.001)
Sanda et al. (2016) [61]	Peri-implant bone	Implants covered by pig’s oral mucosa, and implants embedded into dead pig’s jawbone	Dental swept-source OCT system (Prototype 2, Panasonic, Saijo, Ehime, Japan); 1330 nm central wavelength laser source with 100 nm bandwidth at a scanning rate of 30 kHz	1. Implant surface could be clearly visualized if the mucosal thickness covering the implant was <1 mm 2. Clear images of the implant surface and peri-implant bone could not be obtained when the implants were embedded into the jawbone

## Data Availability

Not applicable.

## Abbreviations

The following abbreviations are used in this manuscript:

OCT	Optical coherence tomography
3D	Three-dimensional
CBCT	Cone-beam computed tomography
IU	Intraoral ultrasound
TD-OCT	Time-domain OCT
FD-OCT	Fourier-domain OCT
SD-OCT	Spectral-domain OCT
SS-OCT	Swept-source OCT
LC-OCT	Line-field confocal OCT
OCTA	OCT angiography
CBL	Crestal bone level
NAFLD	Non-alcoholic fatty liver disease
AI	Artificial intelligence
ML	Machine learning
CEJ	Cementoenamel junction
GT	Gingival thickness
MRI	Magnetic resonance imaging
IL-1β	Interleukin-1β
CNNs	Convolutional neural network
NLP	Natural language processing
